# Editorial: Genetic Mechanisms of Biomarkers in Schizophrenia, Bipolar Disorder and Depression

**DOI:** 10.3389/fpsyt.2021.736055

**Published:** 2021-08-12

**Authors:** Yiguo Tang, Hongsheng Gui, Sarah Tarbox-Berry, Shaohua Hu, Xiancang Ma, Qiang Wang

**Affiliations:** ^1^Mental Health Center and Psychiatric Laboratory, State Key Laboratory of Biotherapy, West China Hospital of Sichuan University, Chengdu, China; ^2^West China Brain Research Center, West China Hospital of Sichuan University, Chengdu, China; ^3^Behavioral Health and Psychiatry Research, Henry Ford Health System, Detroit, MI, United States; ^4^Department of Neurology, School of Medicine, Wayne State University, Detroit, MI, United States; ^5^Department of Psychiatry, School of Medicine, Yale University, New Haven, CT, United States; ^6^Department of Psychiatry, First Affiliated Hospital, Zhejiang University School of Medicine, Hangzhou, China; ^7^Department of Psychiatry, The First Affiliated Hospital of Xi'an Jiaotong University, Xi'an, China; ^8^Center for Brain Science, The First Affiliated Hospital of Xi'an Jiaotong University, Xi'an, China; ^9^Clinical Research Center for Psychiatric Medicine of Shaanxi, The First Affiliated Hospital of Xi'an Jiaotong University, Xi'an, China

**Keywords:** genetic mechanism, biomarker, bipolar disorder, depression, schizophrenia

## Introduction

Mental illnesses, one of seriously intractable chronic diseases, are exerting pernicious effect upon people's daily life. Most of the patients with psychiatric disorders are often unable to fulfill their social and occupational functions. Currently, symptom-based criteria of Diagnostic and Statistical Manual of Mental Disorders (DSM) or International Classification of Diseases (ICD) are indispensable to the diagnosis of mental disorders, in which there is no biological index clearly delineating the boundary between abnormality and normality of mental health. Moreover, considering clinical heterogeneity, genetic background underlying the same clinical symptoms can be very different. Thereby, delving into genetic mechanisms beneath is of vital importance for figuring out the pathophysiological changes and monitoring the disease progression and treatment response.

Although the exact etiology of psychiatric diseases still remains unclear, a large proportion of susceptibility to mental illnesses can be explained by genetic factors (1). Each genetic variation slightly increases the susceptibility, but the cumulative effect of the variations, particularly in the presence of interaction with environmental risk factors, may serve as a strong trigger for mental dysfunction. In the arena of genetic analyses, genome-wide association study (GWAS) is widely applied to detect the genetic polymorphism of individuals across the whole genome, hence can obtain millions of genotypes to be analyzed along with phenotypes at the population level (2). By virtue of sample statistics and their statistical significance (i.e., *p*-value), the genetic variations most likely to affect traits are picked out and the inferred susceptibility loci related to trait variations are then identified. Based on GWAS, the polygenic risk score (PRS) was later developed to quantify the cumulative effect of multiple genes or loci by switching genomic information into a comprehensive score measuring susceptibility to diseases (3). And over the past years, PRSs have been demonstrated to be a promising biomarker by a large amount of evidence. Moreover, neurocognitive functions and neuroimaging as quantitative characters (in other words, endophenotype) have also found their ways into GWAS analyses (4).

Studies within this Research Topic adopted an array of methods to assess biomarkers and molecular genetics in schizophrenia, bipolar disorder, and major depression disorder (MDD), mainly touching upon the following scopes:

(1) detection of genome variations;
(2) integrated analysis of genetics and biomarkers;
(3) exploration of the biological subtypes;
(4) study of biological markers for cross-diseases diagnosis;
(5) identification of the structural and functional brain abnormalities.

## Detection of Genome Variations

Numerous studies have provided consistent evidence that genetic factors are involved in the pathogenesis of mental disorders. The heritability of schizophrenia, bipolar disorder, and MDD are 0.81, 0.75, 0.37, respectively (5); and this indicates that there is a significant genetic component to their etiology, especially for the former two. However, since there are no completely consistent results regarding to the exact genetic factors, identifying susceptibility loci from numerous candidate genes is still a huge ongoing challenge.

Levchenko et al. unveiled associations of alleles of NC_000008.11:g.32614509_32614510del, rs61731109, and rs10508649 (*NRG1* and *PIP4K2A*) with antidepressant treatment response, of alleles of rs35641374 (*NRG1*) and rs10508649 (*PIP4K2A*) with time to recurrence of depressive and manic or mixed episodes among patients with bipolar disorder, and of allele A of rs2248440 (*HTR2C*) with depression severity. In the systematic review by Rovný et al., two polymorphisms related to gating function (*HTR2A* rs6311 and *TCF4* rs9960767) were reported to be associated with schizophrenia at a meta-analytic or genome-wide level, providing further insight into the etiopathogenetic links between genetic variation, gating efficiency, and schizophrenia. According to the primary etiological hypotheses of schizophrenia, glutamate is believed to be a contributor to the onset of schizophrenia. However, Li W. et al. identified that genetic variations of *SLC1A1*, which were generally considered as a critical role in regulating the glutamatergic system, were not the susceptibility biomarkers for schizophrenia, but instead for psychopathology symptoms.

## Integrated Analysis of Genetics and Biomarkers

By integrating the information of multiple genetic susceptibility loci with other potential biomarkers, the capacity for predicting, screening, and intervening high-risk populations prior to the onset of mental diseases, which is the centerpiece of precise prevention of complex diseases, can be greatly enhanced.

Tao et al. first reported associations between cognitive impairment, insulin resistance (IR), and oxidative stress in the first-episode untreated schizophrenics, suggesting that IR may also be a peripheral biological marker of cognitive dysfunction in schizophrenics. A systematic review of potential biomarkers of MDD by Shao and Zhu elaborated upon the associations between monoamine signaling deficits, detrimental personality traits and MDD. As mentioned by Levchenko et al., protein interactions were also involved in the pathogenesis of mood disorders, hence proteomic biomarkers have attracted much attention for its involvement in the occurrence and development of various psychiatric disorders. Wu et al. first established the relationship between the *AQP4* polymorphisms and the risk of schizophrenia in the Southern Chinese Han population. Fu et al. reported a positive association between brain-derived neurotrophic factor (BDNF) polymorphism rs6265 and schizophrenia. In their cis-mQTL (Methylation Quantitative Trait Loci) analysis, an association of rs6265 with various methylation loci surrounding BDNF was detected, further supporting the promising role of BDNF-related methylation in the pathophysiology of schizophrenia. To hammer away at the proteomic biomarkers that can differentiate patients with schizophrenia, bipolar disorder, and MDD and predict the transition of the high-risk group to mental illness, Lee et al. have initiated the Seoul Pluripotent Risk for Mental Illness (SPRIM) study. Moreover, environmental factors, such as intestinal microorganisms, also exert a prominent influence on the etiology of mental illnesses. Cai et al. discovered that mGluR5^−/−^ mice were susceptible to despair-like behavior and the systemic knockout of mGluR5 did not affect the gut microbiota or inflammatory.

## Exploration of Biological Subtypes

Exploration of biological subtypes creates a fertile climate for the research domain criteria. Wang et al. reported that the occurrence and development of depressive symptoms in schizophrenia might be influenced by SNP rs3758391 through the dysregulation of *SIRT1* mRNA expression. In the study of genetic variations of *SLC1A1* mentioned above, Li W. et al. also found five SNPs (rs7032326, rs7860087, rs2039291, rs4742007, and rs301430) related to subtype symptoms. In addition, neuroimaging techniques and neuroelectrophysiology also provide further insight into biological subtypes of psychiatric illnesses.

## Study of Cross-Disease Biological Markers

Comorbidities of different psychiatric disorders are prevalent, while some of these clinical sharing can be explained by an overlapping genetic basis. Recent post-GWAS analyses, as examples indicated from FUMA, Sherlock, and SMR can be further conducted to predicate candidate genes and identify the pleiotropic genes which contribute to various traits simultaneously. By adopting those *in silico* approaches above, Liu et al. revealed 21 potential pleiotropic genes and three biological pathways highly likely to be shared between schizophrenia and cardiometabolic disease. Ullah et al. established for the first time the association of highly recurrent copy number variations with schizophrenia and premenstrual dysphoric disorder. Cao et al. not only revealed three novel microRNAs (hsa-miR-208b-3p, hsa-miR-494-5p, and hsa-miR-208a-3p) potentially contributing to schizophrenia, but also suggested that higher cardiovascular mortality and lower odds of glioma in schizophrenic patients could be explained by the sharing of regulatory networks between schizophrenia and other pathologies.

## Identification of the Structural and Functional Brain Abnormalities

Structural and functional brain abnormalities identified by neuroimaging techniques and neuroelectrophysiology, such as fMRI, DTI, and EEG, further enhancing the understanding of neural mechanisms of psychiatric diseases.

Li Z. et al. evaluated changes in brain gray matter structure by measuring cerebral cortex thickness and subcortical gray matter volume, and reported that *MTHFR* C677T polymorphism may be involved in the dysfunction of limbic–cortical–striatal–pallidal–thalamic (LCSPT) circuits mediating emotion processing. As mentioned above, neuroimaging techniques and neuroelectrophysiology also contribute to the exploration of the potential biomarkers of biological subtypes. Auditory verbal hallucinations (AVH), a core feature of schizophrenia, refer to hearing voices without external stimulation in the awake state (6, 7). Such symptoms not only occur in 60–90% schizophrenics (8, 9), but also feature in a range of other psychiatric disorders. Sun, Fang, Shi et al. pinpointed that inhibitory control was impaired in schizophrenia patients, and worse inhibitory top-down control might contribute causally to the onset of AVH, echoing the most influential hypotheses of AVH (10). And by taking advantage of EEG, they also discovered that AVH in schizophrenia may be related to neuropathological abnormalities in frontal-central brain regions. Additionally in another study lead by the same group, Sun, Fang, Peng et al. found that AVH patients showed higher activity level in the resting-state and may have impaired higher-order auditory expectations in the task-related state, speculating that the occurrence of AVH may occupy certain brain resources and compete for brain resources with external auditory stimuli. Furthermore, through resting-state fMRI scans, Gao et al. found abnormal connections related to auditory, speech, and memory circuits, including the STG, Wernicke's area, Broca's area, and hippocampus, in schizophrenia patients with AVH.

## Conclusion

In a nutshell, through delving into the genetic mechanisms of biomarkers in schizophrenia, bipolar disorder, and MDD, a better understanding of the etiology of these mental disorders as well as comorbidity comes into being. With more development in this area of reseach, psychiatrists will be able to make more appropriate decisions in diagnosis, treatment selection and prediction of disease course.

## Author Contributions

All authors conceived and developed the presented ideas and contributed to the final manuscript equally.

## Conflict of Interest

The authors declare that the research was conducted in the absence of any commercial or financial relationships that could be construed as a potential conflict of interest.

## Publisher's Note

All claims expressed in this article are solely those of the authors and do not necessarily represent those of their affiliated organizations, or those of the publisher, the editors and the reviewers. Any product that may be evaluated in this article, or claim that may be made by its manufacturer, is not guaranteed or endorsed by the publisher.
